# Single-cell discovery of the scene and potential immunotherapeutic target in hypopharyngeal tumor environment

**DOI:** 10.1038/s41417-022-00567-x

**Published:** 2022-12-02

**Authors:** Chen Lin, Yanguo Li, Yidian Chu, Yaqin Lu, Zhengyu Wei, Hongxia Deng, Shanshan Gu, Qi Ding, Zhisen Shen, Qi Liao

**Affiliations:** 1 The Affiliated Lihuili Hospital, Ningbo University, Ningbo, China; 2 School of Medicine, Ningbo University, Ningbo, China; 3 Institute of Drug Discovery Technology, Ningbo University, Ningbo, China; 4 The Ningbo Diagnostic Pathology Center, Ningbo, China

**Keywords:** Head and neck cancer, Biomarkers

## Abstract

Hypopharyngeal carcinoma is a cancer with the worst prognosis. We constructed the first single-cell transcriptome map for hypopharyngeal carcinoma and explored its underlying mechanisms. We systematically studied single-cell transcriptome data of 17,599 cells from hypopharyngeal carcinoma and paracancerous tissues. We identified categories of cells by dimensionality reduction and performed further subgroup analysis. Focusing on the potential mechanism in the cellular communication of hypopharyngeal carcinoma, we predicted ligand-receptor interactions and verified them via immunohistochemical and cellular experiments. In total, seven cell types were identified, including epithelial and myeloid cells. Subsequently, subgroup analysis showed significant tumor heterogeneity. Based on the pathological type of squamous cell carcinoma, we focused on intercellular communication between epithelial cells and various cells. We predicted the crosstalk and inferred the regulatory effect of cellular active ligands on the surface receptor of epithelial cells. From the top potential pairs, we focused on the *BMPR2* receptor for further research, as it showed significantly higher expression in epithelial cancer tissue than in adjacent tissue. Further bioinformatics analysis, immunohistochemical staining, and cell experiments also confirmed its cancer-promoting effects. Overall, the single-cell perspective revealed complex crosstalk in hypopharyngeal cancer, in which *BMPR2* promotes its proliferation and migration, providing a rationale for further study and treatment of this carcinoma.

## Introduction

Hypopharyngeal carcinoma (HC), which accounts for approximately 3% of all head and neck cancers [1], is a relatively rare type of cancer. The main pathological type is squamous cell carcinoma [2], and the common level of differentiation is low. The overall five-year survival rate for hypopharyngeal carcinoma is reported to be approximately 30–35% [3], which is a cancer with the worst prognosis among head and neck cancers (HNC). Anatomically, the hypopharynx is defined by its subsite, including the lateral pharynx, the posterior pharyngeal wall, the piriform sinus, and the posterior annular area leading to the entrance of the esophagus [4]. The symptoms and signs of cancer are not obvious in the early stage, but patients will experience symptoms such as hoarseness and dyspnea in the late stage, when hypopharyngeal cancer is often diagnosed [3, 5]. Contrast-enhanced computed tomography (CT) and magnetic resonance imaging (MRI) are the main diagnostic studies for evaluating primary tumor [6], regional lymph node, and cartilage invasion of hypopharyngeal cancer. Furthermore, this disease is prone to invasion and metastasis, and cervical lymph node metastasis can occur in the early stages [7]. Therefore, to improve the survival and quality of life of patients, we need to identify more active treatments.

Hypopharyngeal cancer surgery will have a significant impact on the laryngeal function of patients, including pronunciation, breathing, and diet, and greatly reduces the quality of life of patients [8]. Currently, nonoperative treatment is usually the first choice for HPV-positive and negative hypopharyngeal carcinoma, but surgery can be used if the patient does not meet the indication of radiotherapy or is in an advanced stage with laryngeal cartilage invasion (Stage T4) or laryngeal dysfunction [9]. Although the quality of life of patients with hypopharyngeal cancer has improved further with the increase of laryngeal function-preserving therapy, the survival rate has not improved significantly.

Single-cell RNA sequencing [10] (scRNA-seq) is a new technique that involves the amplification and sequencing of the full transcriptome at the single-cell level. Its basic principle is to isolate a single cell by amplifying trace amounts of RNA, and then obtain the expression profile of a single cell by high-throughput sequencing, identify cell types, analyze the spatiotemporal course of cell development, and calculate the communication and regulatory network between cells. Compared with traditional multicellular sequencing methods, single-cell transcriptome sequencing greatly retains heterogeneous information between tumor cells [11, 12]. Phenotypic heterogeneity such as immune characteristics, growth rate, and invasive ability eventually lead to differences in sensitivity to different antineoplastic drugs or differences in radiosensitivity [13]. In recent years, single-cell sequencing technology has played an important role in many research fields and is of great significance for early diagnosis, tracking, and personalized treatment of cancer [14–16].

In this study, we analyzed and compared the tumor microenvironment of hypopharyngeal carcinoma by single-cell sequencing and constructed the first single-cell transcriptome map of this cancer. The transcriptional states of these cancers and paracancerous cells are described, with an emphasis on the analysis of cellular crosstalk that is potentially associated with tumor progression. Furthermore, our analysis defined the gene *BMPR2* as a potential clinical target and further confirmed that it acts as an oncogene to promote the proliferation and migration of hypopharyngeal cancer cells at the cellular level. In summary, our objective was to stimulate new research and provide a basis for promoting the early diagnosis and treatment of hypopharyngeal carcinoma through single-cell analysis.

## Materials and methods

### Sample collection

We collected histological samples from two patients with hypopharyngeal squamous cell carcinoma (including two groups of cancer tissues HC1, HC2, and their adjacent tissues PT1, PT2). Both patients signed consent forms at the Li Huili Hospital of Ningbo Medical Center, and the study of the project was approved by the hospital ethics committee (KY2020PJ191). After surgical interventions for the hypopharyngeal tumor, the head and neck otorhinolaryngologist collected the tumor tissue and paracancerous tissue of the patient, which was confirmed by rapid intraoperative pathological sections. All freshly resected biopsies were used for pathology and single-cell sequencing. Patients’ clinical information is as follows (Table 1).

**Table. 1:** Patients’ clinical information.

Patient	Age	Stage	Grade	Tumor size	Tobacco	Alcohol	Surgery
1	66	T2N2bM0	IVA	The largest diameter of 1.7 cm	+	+	Tracheotomy + Left lymph node dissection + Partial hypopharyngeal resection + Laryngeal function reconstruction
2	66	T4N1M0	IVA	5.0 cm × 4.5 cm × 1.7 cm	+	+	Total laryngectomy + Subtotal resection of hypopharynx + Bilateral lymph node dissection + Left lateral thigh free myocutaneous flap repair of hypopharynx

### Pretreatment before sequencing

The fresh tissue sample was divided into portions of 2–4 mm, washed with DPBS 2–3 times, then stored in a preservation solution at 4 °C, and transported to the laboratory on ice. According to the instructions of the Human Tumor Dissociation Kit (MACS 130-095-929), each piece of tissue was digested and subjected to dissociation at 37 °C for 30 min in a dissociation solution containing RPMI 1640 medium, 0.35% collagenase IV5, 2 mg/ml papain, 120 Units/ml DNase. The resulting cell suspension was filtered by passing through a 70-μm stacked cell strainer and centrifuged at 300 × *g* for 5 min at 4 °C. Resuspend cell pellet in 100 ul 1× PBS. Then 1 ml 1× red blood cell lysis buffer (MACS 130-094-183, 10×) was added and incubated on ice for 2–10 min to lyse the remaining red blood cells. After incubation, the suspension was centrifuged at 300 × *g* for 5 min at room temperature. Remove dead cells using Miltenyi ® Dead Cell Removal Kit (MACS 130-090-101) after 100 μl Dead Cell Removal MicroBeads (MACS 130-090-101). Then resuspended and centrifuged as before (repeat twice). Finally resuspended in 50 μl of 1× PBS. The overall cell viability was confirmed by trypan blue exclusion, which needed to be above 85% and single-cell suspensions were adjusted in concentration to 700–1200 cells/μl. After completing the tissue digestion and single-cell suspension preparation, as indicated by the 10×Genomics pre-sequencing needed, we followed by cell sorting, reverse transcription, and amplification.

### Single-cell RNA-seq data processing

Single-cell suspensions were loaded to 10x Chromium to capture 5000 single cells according to the manufacturer’s instructions for the 10X Genomics Chromium Single-Cell 3’ kit (V3). The following cDNA amplification and library construction steps were performed according to the standard protocol. Libraries were sequenced on an Illumina NovaSeq 6000 sequencing system (paired-end multiplexing run,150 bp) by LC-Bio Technology co. ltd. (Hangzhou, China), at a minimum depth of 20,000 reads per cell. The software Cell Ranger (Version 3.1.0) [17] was used to process the raw data. It blasted the FASTQs data output by 10×Genomics Illumina sequencing to the human Ensembl genome GRCh38/GRCm38 reference genome. After the quantitative sequence data were preliminarily processed and counted with Cell Ranger, the data were loaded into Seurat (Version 3.1.1) for further dimensionality reduction, clustering, and analysis. We used the scDblFinder package for dual cell data filtering and to eliminate the interference by mitochondria, blood cells, and ribosomal genes in the process of quality control. The data filtering and quality control standards (Fig. S1A, B) were as follows: (1) all genes remained have to express at least in 1 cell; (2) the minimum number of genes expressed in each cell was set at 700; (3) the lowest expression count value of each gene was 600; (4) The average number of genes for each UMI (log10GenesPerUMI) ≥0.78. Unique molecular identifiers (UMI) is the adding unique tag sequences to each fragment of the original sample genome after interruption.; and (5) the proportion of mitochondrial gene expression in single cells was set as 30%. We then used the SCTransform method to further standardize the data and calculate the expression value.

### Cell types and identification of subtypes

To visualize the data, we used Seurat software to further reduce the dimension of 17,599 cells. The Harmony [18] algorithm was carried out to perform principal component analysis (PCA) and nonlinear dimensionality reduction of expression values. The first 35 principal components were selected from the results of the PCA analysis for subsequent clustering and clustering analysis. After nonlinear dimensionality reduction via t-SNE and UMAP, single-cell consensus clustering (SC3) was used for unsupervised clustering. The preliminary results of the clustering were visualized by the t-SNE and UMAP packages, and then cell identification was achieved using the SingleR package and further manual identification of the marker gene. Finally, we defined 25 cell clusters and seven cell types. Subsets of all kinds of cells are re-clustered, as described above.

### Functional annotation and pathway enrichment

Kyoto Encyclopedia of Genes and Genomes [19] (KEGG) is a database resource used to understand the advanced functions and pathways of biological systems. Pathways with a Q value ≤0.05 were considered significantly enriched. The functional annotation of Gene Oncology [20] (GO) is based on the gene ontology database, including biological processes, cellular components, and molecular functions. The Fisher exact test was used to select important categories and GO terms with a Q value <0.05 were considered important. Both tools were implemented in the R tool. *Q* value, the adjusted *P* value by the FDR method.

### Cell communication

Cell-cell communication mediated by the ligand-receptor complex plays a key role in a variety of biological processes, such as tumorigenesis and tumor development, and peripheral inflammation [21]. We compared differences in the expression of ligand and receptor genes and unraveled the communication between hypopharyngeal carcinoma and its adjacent cell types using the iTALK package. In addition, we combined cell expression data with known signals and gene regulatory networks through NicheNet packages, and further analyzed the communication between two specific subsets of interest to predict their ligand-receptor interactions. Applying NicheNet to the number of tumors and immune cell microenvironments, we inferred active ligands and gene regulation on interacting cells.

### Bioinformatics analysis methods

Sequencing data and the clinical information of 545 patients with head and neck cancer included in TCGA were downloaded for expression analysis and statistical analysis of clinical data. Cox analysis was based on packages, including “ survminer “, “survival”, and “ggplot2” for regression analysis and composition of TCGA clinical data. The miRNA sequencing dataset GSE117558 of the hypopharyngeal carcinoma dataset, complete with the associated survival data, were downloaded from the GEO database, and the differential expression analysis was carried out using GEO2R; miRNA with significant differences (*P* < 0.05) were obtained. Targetscan7.1 predicts the miRNA of the target gene. The miRNA that targets the gene and is related to survival can be obtained by the intersection of the above datasets.

### Immunohistochemical

The EnVision two-step method was used for immunohistochemical staining of the paraffin-fixed samples. Samples were prepared by incubating tissue slices in ethylenediamine tetraacetic acid (EDTA) antigen repair fluid at 100 °C for 20 min. They were then cooled to room temperature. The anti-BMPR2 antibody was purchased from Abcam Company (Cambridge, UK) and used at a working concentration of 1:100. The second antibody, DAB + chromogen and its substrate buffer were all purchased from Dako (Glostrup, Denmark). The results of immunohistochemical staining were evaluated by two pathologists using a double-blind method. We then further quantified our results using the ImageJ IHC Profiler tool.

### Cell culture

The normal hypopharyngeal cancer cell line FaDu was purchased from the BeNa Culture Collection (BNCC, Xinyang City, Henan Province, China). Cells were cultured in a 1:1 mixture of Dulbecco’s modified Eagle’s Medium (DMEM, HyClone, Logan, UT, USA), supplemented with 10% fetal bovine serum (FBS, GibcoTM), and 1% penicillin/streptomycin (P/S) (Gibco, Grand Island, NY, USA) and maintained under standard culture conditions (37 °C, 5% CO_2_, >90% relative humidity).

### Transfection

Cells were seeded cells in culture plates and transfected with 0.08 μM si_BMPR2 using the Lipofectamine 2000 transfection reagent (Life Technologies, Carlsbad, CA, USA) when cells reached a confluency of 60–80%. The sequences of the *BMPR2* inhibitor and its negative control used were 5′-GGGACAUAAAUCUUGUAAATT-3′ and 5′- UUUACAAGAUUUAUGUCCCTT-3′, respectively. The RNA oligo was designed and synthesized by GenePharma (Shanghai City, China).

### RNA pretreatment and reverse transcription

Total RNA in cells was extracted with the TRIzol reagent (Invitrogen, Karlsruhe, Germany). We measured the RNA quality with a SmartSpec Plus UltraMicro Spectrophotometer (Bio-Rad, Hercules, CA, USA). The acceptable purity of the RNA was defined by the A260/A280 values of 1.8–2.1. RNA was stored at −80 °C until further use.

The manufacturer’s protocol was followed for cDNA preparation. The rtStar™ First-Strand cDNA Synthesis Kit (Arraystar) was then used to synthesize cDNA for the detection of *BMPR2* expression by with qRT-PCR. The forward and reverse primers were 5′-TTAGTGACTTTGGACTGTCCATGAG-3′ and 5′-TCTAGCACTTCTGGTGCCATATATCT-3′.

### Cell proliferation

The 5‑Ethynyl‑2′‑deoxyuridine (EdU) assay was used to measure cell proliferation. Forty-eight hours after transfection with siRNAs (Fig. S3D), cells were fixed with 10 μM EdU for 2 h according to BeyoClick™ EdU Cell Proliferation Kit with Alexa Fluor 594 (Beyotime, China). Then, cells were fixed with 4% polyformaldehyde in PBS at room temperature for 30 min, washed, and subsequently incubated with Enhanced Immunostaining Permeabilization Solution (Beyotime, China) for 10 min. After washing for times, the cells were incubated with Click Addictive Solution for 30 min under dark conditions. Finally, dyed nucleus with 1X Hoechst 33343 solution for 10 min. Fluorescence microscopy was performed in five randomly selected fields to analyze proliferation rates. Blue, Hoechst 33343 staining of nuclei with FaDu cells; red staining of EdU with proliferating cells; overlay, the percentage of proliferating cells.

The Cell Counting Kit 8 (CCK-8) (Dojindo Molecular Technologies, Kumamoto, Japan) was used to assay the proliferation of FaDu cells. Specifically, 48 h after transfection with the *BMPR2* inhibitor, 4000 cells in 100 μL were seeded per well of a 96-well plate. Each treatment group was tested with six replicates. After incubating the cells in a 37 °C incubator with 5% CO_2_ for 24 h, a total of 10 μL of CCK-8 solution was added to each well, and FaDu cells were measured after incubation for another 3 h. The absorbance was measured at 450 nm with a microplate reader (SpectraMax M5, Molecular Devices, CA, USA).

### Transwell assay

The transwell assay was used to measure the cell migration of FaDu cells. Transfected cells were first incubated in a 37 °C, 5% CO_2_ incubator for 48 h and then resuspended in Opti-MEM I Reduced serum medium (Gibco). A total of 40,000 cells (200 μL) were added to the upper chamber of a Transwell insert (Costar, Corning, NY, USA), and 500 μL of DMEM medium containing 12% FBS was added to the lower chamber. After the cells were cultured at 37 °C, in a 5% CO_2_ incubator for 24 h, the cells were fixed and stained with paraformaldehyde fixation and crystal violet dye. Then the migrating cells were counted.

## Results

### Single-cell overview of different cell types in hypopharyngeal carcinoma

**Fig. 1:**
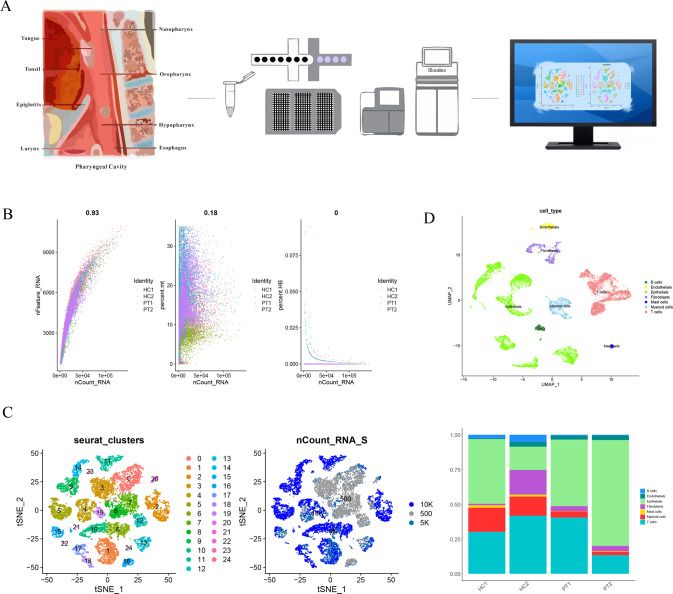
Overview of hypopharyngeal carcinoma. **A** Workflow of hypopharyngeal carcinoma from the sampling site to the 10X Genomics single-cell sequencing and analysis; **B** Scatter plots of nFeatures/nCount value and mitochondrial and erythrocyte ratio after quality control; **C** t-SNE plots visualized cell clusters and viability; **D** UMAP plot and distribution histogram of the identified cell types.

We performed a single-cell analysis of hypopharyngeal carcinoma and paracancerous tissues of two subjects (Fig. 1A). After the quality evaluation of single-cell sequencing data and double-cell filtration, the resulting gene expression profiles of the samples showed a strong correlation, indicating that there was no obvious batch effect between the samples (Fig. S1D). After merging the four datasets and further quality control (Fig. 1B), we obtained a cell-gene matrix of 17,599 cells, with an average of 2680 genes detected per cell. Through principal component analysis (PCA) and dimensionality reduction, we divided the cells into a total of 25 different clusters (Fig. 1C) and identified cluster-specific markers.

Clusters 1, 2, 5, 6, 9, 10, 11, 14, 21, and 24 were epithelial cells, clusters 0, 3, 7, 8, and 12 were T cells, cluster 16 consisted of B cells, cluster 20 consisted of Mast cells, clusters 4, 19, and 23 were myeloid cells, clusters 15 and 22 were endothelial cells, and clusters 13, 17, and 18 were fibroblasts. Figure 1D shows each cell type and its respective proportion (Fig. 1D and Fig. S1C).

### Heterogeneity of the hypopharyngeal carcinoma epithelial cell

**Fig. 2:**
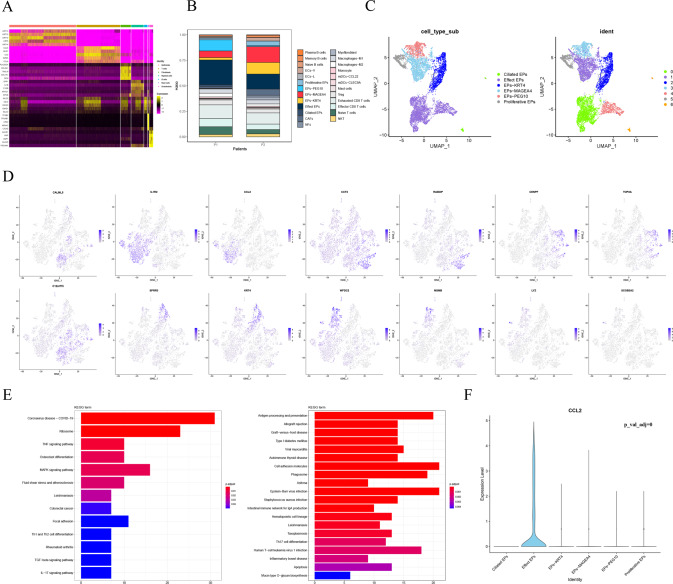
Epithelial cell type in heterogeneous hypopharyngeal carcinoma. **A** Heatmap of significant genes among different cell types; **B** The percentage figure of each cell subtype distribution in different patients; **C** UMAP plots visualized epithelial cell subtypes and clusters; **D** Feature plots of significant markers of six epithelial subtypes; **E** Pathway enrichment of effect Eps components in the epithelial cell, including cluster 0 and cluster 4; **F** *CCL2* is specifically expressed in effect Eps subtype.

There is abundant heterogeneity in hypopharyngeal carcinoma. During cell identification, a total of 8297 epithelial cells were obtained. Of the other nonepithelial cells, including T cells, B cells, mast cells, myeloid cells, endothelial cells, and fibroblasts, a total of 5436, 402, 160, 482, and 1278 cells, respectively, were successively obtained. There were significant differences in gene expression associated with different functions among each cell type. (Fig. 2A). We combined the expression of marker genes with the SingleR prediction package to identify the cell subsets of each cell type (Fig. 2B).

In epithelial cells, seven clusters were obtained for further subgroup identification. Based on the expression characteristics of the marker genes and the prediction of the function of each group of cells, we identified six potential subtypes (Fig. 2C): effector epithelial cells (Effect Eps), *MAGEA4-*specific epithelial cells (EPs-MAGEA4), *KRT4* specific epithelial cells (EPs-KRT4), *PEG10-*specific epithelial cells (EPs-PEG10), proliferative epithelial cells (Proliferative EPs), and ciliated epithelial cells (Ciliated EPs). The significant marker characteristics of six epithelial cell subtypes are shown in Fig. 2D. Among them, we found that the Effect Eps, which has the highest proportion of all cell subsets, was present in almost all of the paracancerous tissue. It defined cluster 0 and cluster 4, in which cluster 0 pathways were enriched in the Ribosome, MAPK signaling pathway, Focal adhesion, and TNF signaling pathway, while cluster 4 was enriched in antigen processing and presentation, allograft rejection, and graft-versus-host disease pathways (Fig. 2E). Many of the pathways involved are related to the inflammatory response. Further, this subgroup of cells specifically expressed the *CCL2* gene, which has been confirmed to be involved in the regulation of the inflammatory response, humoral immune response, and apoptosis (Fig. 2F). Most of the epithelial cells in the Eps-PEG10 subsets were located in tumor tissues. As shown in Fig. S1E, through functional annotation and pathway enrichment, we found that its significant markers were enriched in biological functions such as mitochondrial translational elongation, mitochondrial translational termination, translational termination, and mitochondrial translation. Most of the functions enriched by the GO functional annotation were related to mitochondria. Gao [22] et al. proposed the important role of mitochondrial dynamics in the activation of the immune cell response. Our analysis of the function of this type of tumor epithelial cells also suggests that mitochondrial kinetics may be a potential target for new drugs in antitumor immunotherapy.

### Tumor microenvironment of hypopharyngeal carcinoma and its complex intercellular communication network

**Fig. 3:**
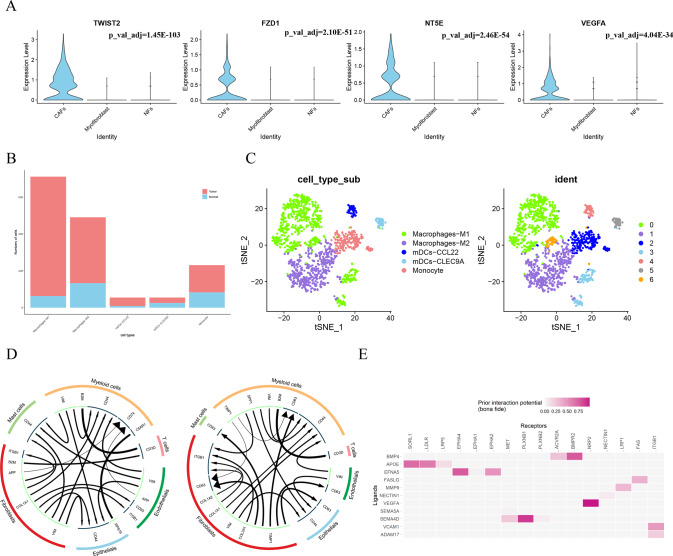
Complex compositions of the hypopharyngeal carcinoma microenvironment. **A** Violin plots of specifically expressed oncogenes in CAFs; **B** Proportion of cell numbers of myeloid cell subtypes; **C** t-SNE plots visualized myeloid cell subtypes and clusters; **D** Complex intercellular crosstalk of patient 1 and patient 2; **E** Heatmap of top ligand-receptor pairs while setting epithelial cells as the message receiver.

The component of the tumor microenvironment in hypopharyngeal carcinoma is inseparable from various types of cells. In addition to epithelial cells, we also analyzed subsets of other nonepithelial cells. In the subsets of fibroblasts, cancer-associated fibroblasts (CAFs) specifically express oncogenes such as *NT5E* and *FZD1* (Fig. 3A). Subsequently, in the re-clustering analysis of myeloid cells, we obtained M1 Macrophages, M2 Macrophages, monocytes, CCL22-specific dendritic cells, and CLEC9A-specific dendritic cells. Unlike most other types of nonepithelial cells, the number of these cells in cancer tissues is higher than that in paracancerous tissues (Fig. 3B, C). The cells we mentioned may play a role in the process of tumor formation and immune resistance.

Furthermore, there is complex intercellular crosstalk between individual cells in hypopharyngeal carcinoma and adjacent tissues, and these interaction networks form an important part of the tumor microenvironment of hypopharyngeal carcinoma. Therefore, exploring the relationship of intercellular communication is helpful in gaining a better understanding and insight into the development and mechanism of hypopharyngeal carcinoma.

The intercellular communication network of the two patients was predicted by the iTALK package. As shown in Fig. 3D, the top ligand pairs of cancer and paracancerous tissues in patient 1 included: VIM-CD44, COL1A1-CD44, COL1A1-ITGB1, B2M-CD3D, APP-CD74, RPS19-C5AR1; patient 2 top ligand pairs include: TIMP1-CD63, COL3A1-ITGB1, COL1A2ITGB1, VIM-CD44, COL1A1-CD44, SPP1-CD44, B2M-CD3D. We found that VIM-CD44, COL1A1-CD44, B2M-CD3D, and the interaction between the ligand COL1A1/COL1A2/COL3A1 and the ITGB1 receptor showed significant benefits in the tumor microenvironment of both patients. Of these, β 2-microglobulin (B2M) is an important subunit of class I major histocompatibility complex (MHC), which plays a substantial biological role in tumorigenesis and immunological activity [23]. At present, corresponding targeted drugs named navumab and pimumab have been put into clinical use for the treatment of several cancer types. Collagen such as COL1A1/COL1A2/COL3A1 can change the resistance of tumor cells by interacting with the ITGB1 integrin, as reported for other cancers [24, 25]. This mechanism has not been mentioned in previous hypopharyngeal cancer studies. Another point is that fibroblasts and myeloid cells are both most significant in these cellular communications networks, hinting that they are the potential to be cellular targets for treating the tumor with drugs.

Since the cancer cell phenotype of hypopharyngeal carcinoma is epithelial cells, we specifically analyzed the binding and regulation of epithelial receptor proteins to ligand proteins of various cell types. Through the nichenet package, we calculated the ligand-receptor network of all cells and epithelial cells and selected the “bonafide” ligand-receptor links described in the literature and not predicted based on PPI for heatmap display (Fig. 3E). The interaction potential scores of the corresponding ligand of the epithelial receptor proteins NRP2, PLXNB1, BMPR2 were the most significant. Previous studies have shown that PLXNB1 can induce epithelial-mesenchymal transformation (EMT) and promote metastasis of head and neck squamous cell carcinoma [26], and NRP2 can promote tumor progression of thyroid papillary carcinoma [27].

### The BMPR4 gene promotes the growth and migration of hypopharyngeal carcinoma

**Fig. 4:**
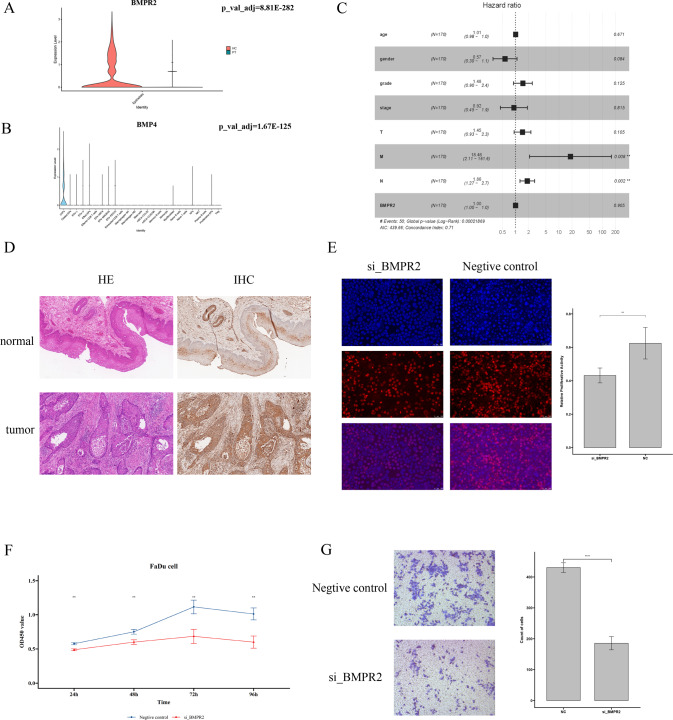
*BMPR2* is of great importance in the tumorigenesis and metastasis of hypopharyngeal cancer. **A** Violin plot of the differential expression of BMPR2 in epithelial cells of tumor tissues and adjacent tissues; **B** BMP4 ligand is expressed in CAFs; **C** Forest plot of Cox analysis of clinical data from *BMPR2* and TCGA clinical data; **D** Immunohistochemistry results of BMPR2 protein expression and location in tumors and adjacent tissues; **E** EdU fluorescence staining result. The red-blue ratio represents cell proliferation activity. Blue, Hoechst 33343 staining of nuclei with FaDu cells; red staining of EdU with proliferating cells; overlay, the percentage of proliferating cells. **F** Line chart of CCK8 experimental results. Method: ANOVA. At least three times for three repeats; **G** Transwell results comparing negative controls and the si_BMPR2-treated hypopharyngeal carcinoma cell line FaDu. Method: *t*-test. Repeat at least three times.

BMPR with a high interaction score has not been studied in head and neck cancer, and there is a significant difference in the expression of *BMPR2* between cancer tissues in the epithelial cell group and adjacent tissues (Fig. 4A). We found that the BMP4 ligand gene corresponding to receptor *BMPR2* is expressed mainly in CAFs, and it has been demonstrated in other cancers types that *BMPR2* is involved in cancer progression and is closely related to the EMT process [28–30]. Otherwise, a small-scale intercellular crosstalk analysis also hints BMP signaling is one of the significant pathways of CAFs and epithelials (Fig. S2). Therefore, *BMPR2* is a potential key gene involved in the progression of hypopharyngeal carcinoma and deserves further analysis and verification regarding the function of this gene.

First, we downloaded head and neck cancer datasets and clinical data files from The Cancer Genome Atlas (TCGA) database. Multivariate analysis of valid clinical data using Cox regression analysis showed in Fig. 4C that there was a significant correlation between *BMPR2* expression and distant cancer metastasis of cancer (*p* = 0.008). In addition, the expression of *BMPR2* was also significantly correlated with lymph node metastasis (*p* = 0.002). Subsequently, we verified whether the gene expressed its protein by immunohistochemical analysis, and the results showed that the gene expressed the BMPR2 protein (Fig. 4D and Fig. S4), whose levels in the hypopharyngeal carcinomatous epithelium was significantly higher than that in the adjacent normal epithelium, and the protein was located in the cytoplasm and cell membrane.

Meanwhile, we cultured the hypopharyngeal carcinoma cell line FaDu. After BMPR2 expression was downregulated by small interference RNA (siRNA), we conducted EdU proliferation assays, CCK8 cell proliferation assays, and the transwell cell migration assay. The results of the cell proliferation assay indicated that the proliferation of hypopharyngeal cancer cells with *BMPR2* knockdown was significantly lower than in the negative control group (Fig. 4E, F). The results of the migration assay showed that the number of migrated cells in the treatment group was significantly lower than that in the negative control group (Fig. 4G). In general, reducing the expression of the *BMPR2* gene can negatively regulate the proliferation and migration capacity of hypopharyngeal cancer cells.

Altogether, the results of the functional assays and the analysis of clinical data from TCGA allows to draw the conclusion that *BMPR2* can promote the growth and metastasis of hypopharyngeal carcinoma. Additionally, we downloaded and analyzed the GSE117558 miRNA sequencing dataset of hypopharyngeal carcinoma and related survival data from the GEO database and intersected significantly different miRNAs with conservative miRNAs upstream of *BMPR2* predicted by Targetscan. Seven survival-related miRNAs that may be involved in the regulation of *BMPR2* were identified (Fig. S3E), which can be further evaluated and verified in future studies.

## Discussion

During the process of tumorigenesis, tumor cells show different phenotypes: EMT, stemness, immune infiltration, invasion, migration, hypoxia, and apoptosis. These phenotypes play an important role in tumor invasion, metastasis, dissemination, treatment, and drug resistance [31]. Although traditional methods provide a large amount of genome or transcriptome data, they cannot reveal the cellular heterogeneity that causes this complexity [32]. The emergence and application of single-cell sequencing technology not only retains the heterogeneous information between different cells in tumor tissue but also overcomes the deficiency of traditional high-throughput sequencing. In recent years, with the emergence of microfluidic, random capture, and in situ labeling, single-cell transcriptome sequencing technology has also been automated and commercialized, and the scale of sequencing has increased exponentially [33].

Currently, the mode of treatment for hypopharyngeal cancer has changed from comprehensive surgery-based treatment to simultaneous radiotherapy and chemotherapy, such as tumor uncontrolled followed by salvage surgery. Although the preservation rate of laryngeal function has greatly improved, the survival rate for hypopharyngeal cancer has not changed significantly [34]. In recent decades, clinicians and researchers have dedicated a significant amount of research to the pathogenesis of hypopharyngeal carcinoma, but the molecular mechanisms responsible for the high degree of malignancy and poor prognosis of hypopharyngeal carcinoma have not been fully elucidated. It is necessary to continue to study the potential molecular mechanism leading to the occurrence and development of hypopharyngeal carcinoma and its poor prognosis and to identify new therapeutic targets. The development of single-cell transcriptome sequencing provides a novel approach and perspective for the study of hypopharyngeal carcinoma, a relatively rare tumor with a poor prognosis.

In this study, hypopharyngeal carcinoma and adjacent samples were sequenced by single-cell sequencing. After analysis, the landscape of human hypopharyngeal squamous cell carcinoma was described at single-cell resolution and seven types of cells were identified: epithelial cells, T cells, B cells, mast cells, myeloid cells, endothelial cells, and fibroblasts. Since the pathological type of the samples was low differentiated squamous cell carcinoma, we further analysed epithelial cells and nonepithelial cells. In the identification of epithelial cells, we found that Effect Eps, the largest subgroup, was mainly distributed adjacent to the cancer tissue and was significantly associated with the inflammatory response. Cancer patients in this study were both in the IVA stage, suggesting that paracancer epithelial cells promote the establishment of an immunosuppressive tumor microenvironment through an inflammatory response to promote the occurrence and development of tumors in this stage. Furthermore, almost all cells of the Eps-PEG10 subset were distributed in cancer tissues, and the tumor-related gene *PEG10* [35] was specifically expressed in this group of cells, further, *PEG10* was significantly enriched in mitochondrial translation-related functions. Recent studies have shown that mitochondrial translation can recognize and destroy cancer cells and virus-infected cells in the immune system and is a significant feature of cytotoxic T cells [36]. Inhibition of mitochondrial translation can damage the sustained killing capacity of cytotoxic T cells. This suggests that may be worthwhile to explore potential therapeutic approaches using immunotherapeutic targeting of mitochondrial dynamics in the antitumor treatment of hypopharyngeal cancer.

Additionally, we predict the interactions between ligand receptors and depict a complex network of cellular crosstalk relationships in hypopharyngeal carcinoma. It shows that myeloid cells and fibroblasts are of great activity in the intercellular communication network. Myeloid cells, the components of the innate immune system, is associated with poor prognosis in a variety of tumor types [37, 38]. There are potential tumor therapeutic targets in myeloid cells, in which research showed that tolerogenic myeloid cells-expressed gene *STAT3* works against the radiotherapy of head and neck cancer [39]. In our study, we found that there is a significant benefit between the main target *B2M* gene and its corresponding receptor gene in the hypopharyngeal carcinoma microenvironment, but this target and the corresponding targeting drugs have not been further studied or applied in head and neck cancer. Moreover, the mentioned significant COL1A1/COL1A2/COL3A1 collagens are able to change the resistance of malignant cells by interacting with the ITGB1 integrin among the network. Next, we set epithelial cells as the signal receivers to further examine the ligand-receptor pairs that interact most significantly with squamous cell populations and explore new potential tumor key genes. NRP2, PLXNB1, and BMPR2 were the most significant genes. Angiogenesis can promote the growth of cancer cells, while both NRP2 and PLXNB1 have been verified to be vascular-associated genes [40, 41]. Fibroblasts are key components in angiogenesis, which may play a critical role in the development of hypopharyngeal cancer. The expression of the *BMPR2* receptor gene between cancer and paracancerous epithelial cells was significantly different between the most significant pairs of ligand receptors. The corresponding ligand BMP4 is also secreted specifically by CAFs (Fig. 4B). Subsequently, through the analysis of TCGA clinical information analysis and cell proliferation and migration experiments, we confirmed that the gene plays a key role in hypopharyngeal carcinogenesis, and then concluded that *BMPR2* promotes hypopharyngeal carcinoma metastasis and progression. It is a potential new target for hypopharyngeal cancer treatment and a potential index for evaluating tumor recurrence and metastasis.

In summary, we constructed the first transcriptome landscape of hypopharyngeal carcinoma, and thus revealed the cell composition of the tumor microenvironment of hypopharyngeal carcinoma and its complex cellular crosstalk relationships. On this basis, we found and verified that the *BMPR2* gene promotes tumorigenesis and migration of hypopharyngeal carcinoma. Our study provides a new perspective for further elucidating the tumorigenesis mechanism and discovering biomarkers and therapeutic targets for hypopharyngeal carcinoma.

## Supplementary information

Supplementary Figure 1

Supplementary Figure 2

Supplementary Figure 3

Supplementary Figure 4

Supplementary figure and table legends

Supplementary Table.1

Code

## Data Availability

The raw data that support the findings of this study are available on request from the corresponding author. The raw data/code file after processing had directly uploaded as Supplementary Table 1 and single_cell_HC_code.txt of the article.
