# Supplementary figures and images for: Single-cell discovery of the scene and potential immunotherapeutic target in hypopharyngeal tumor environment
Source: Cancer Gene Ther. 2022 Dec 2;30(3):462–71. doi: 10.1038/s41417-022-00567-x (PMC10014576; doi:10.1038/s41417-022-00567-x)

A

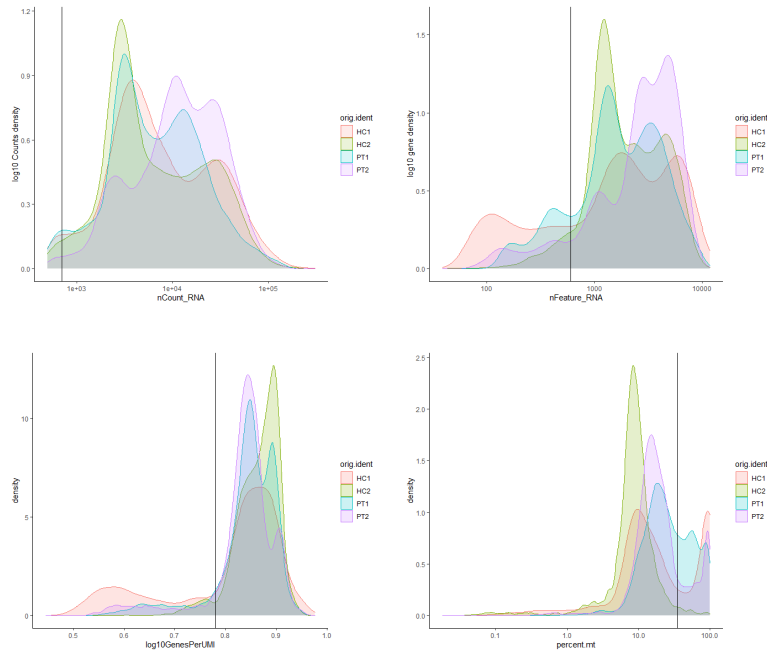

B

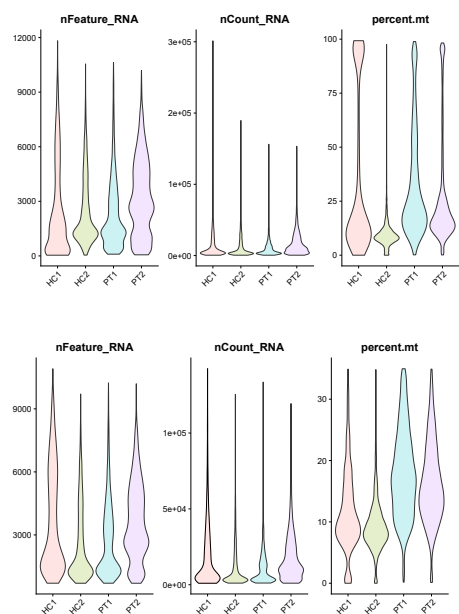

C

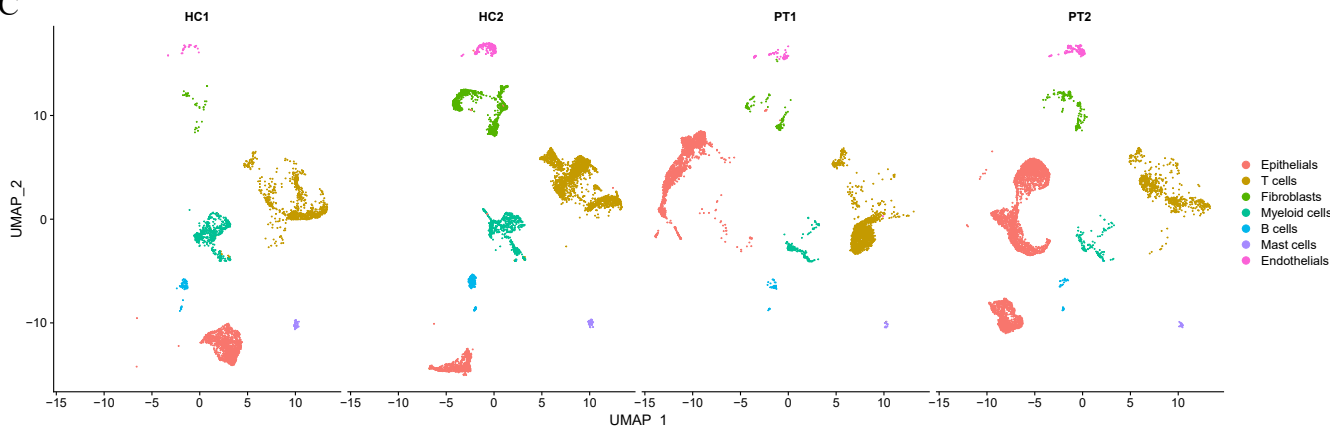

D

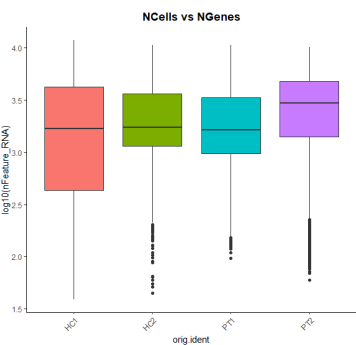

E

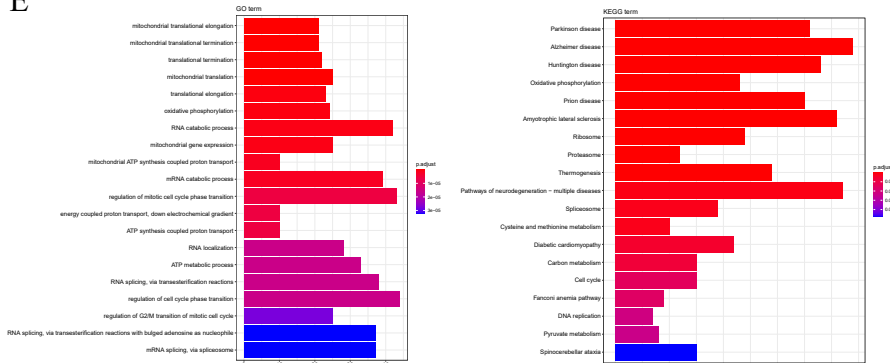

Supplement: Supplementary file 1 — Supplementary Figure 1 [file 41417_2022_567_MOESM1_ESM.pdf]

A

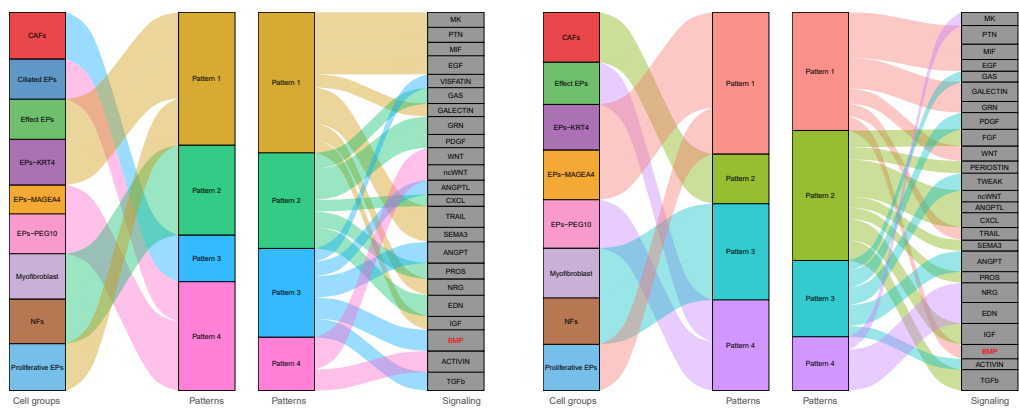

B

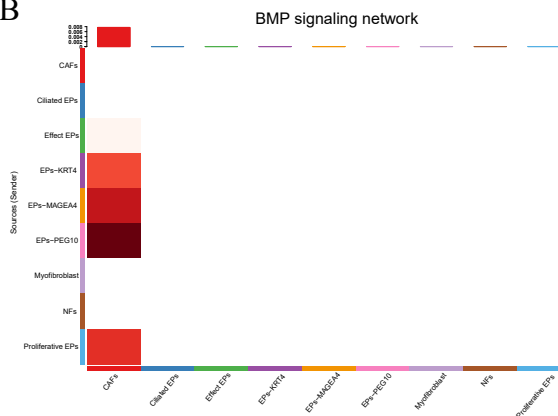

C

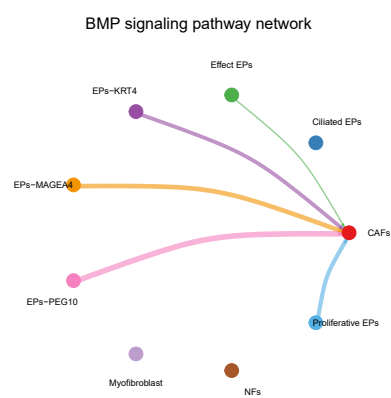

D

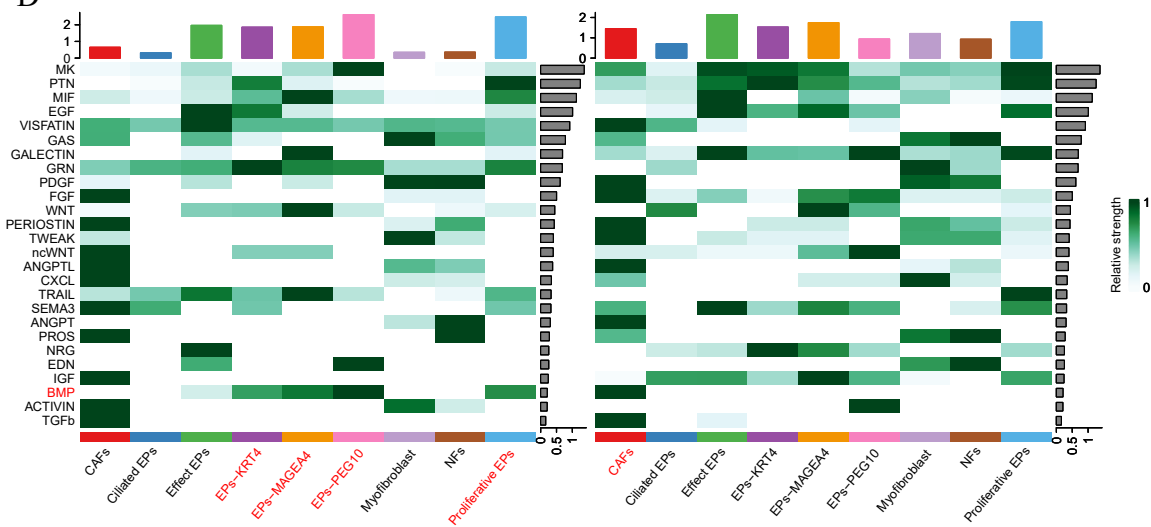

Supplement: Supplementary file 2 — Supplementary Figure 2 [file 41417_2022_567_MOESM2_ESM.pdf]

A

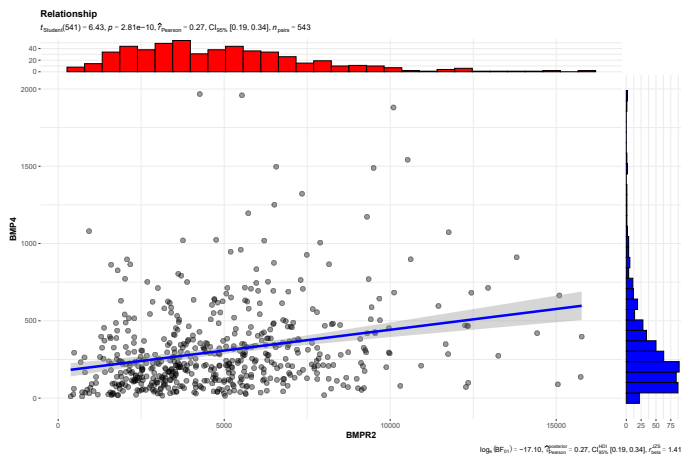

B

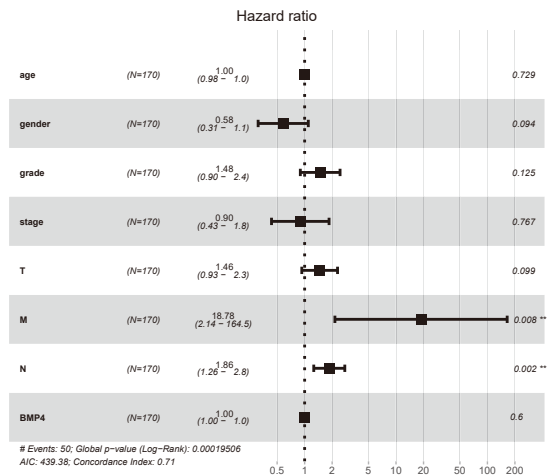

C

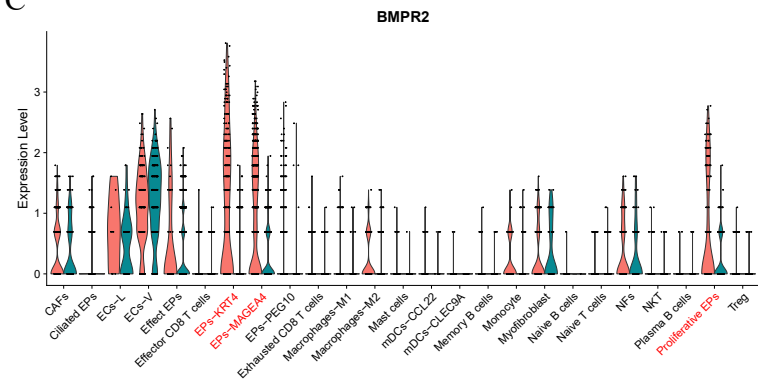

D

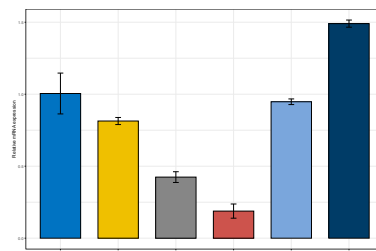

E

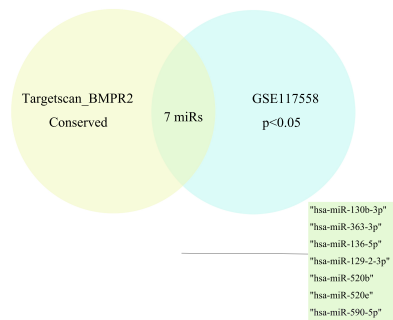

Supplement: Supplementary file 3 — Supplementary Figure 3 [file 41417_2022_567_MOESM3_ESM.pdf]

A

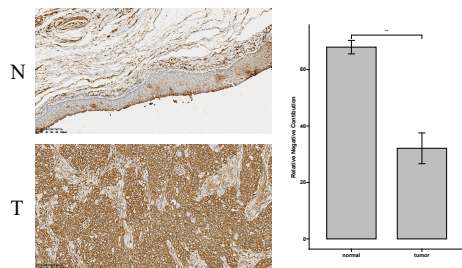

B

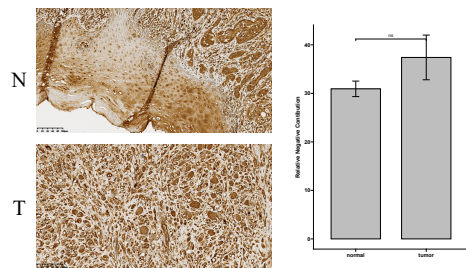

C

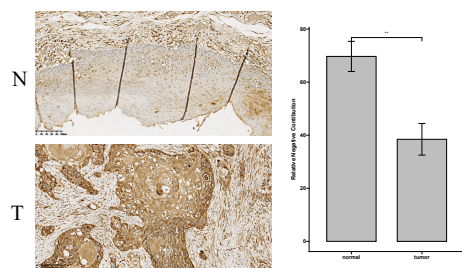

D

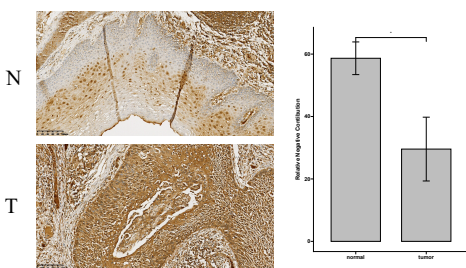

E

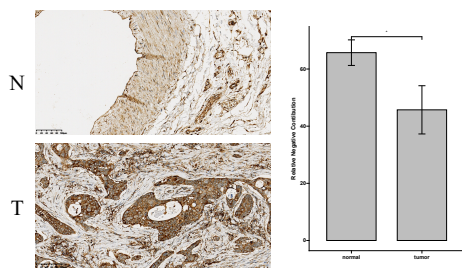

F

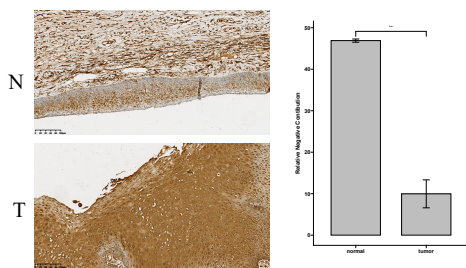

G

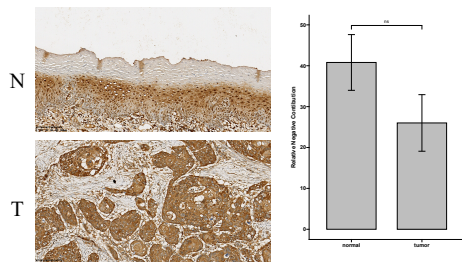

N:normal

T:tumor

Supplement: Supplementary file 4 — Supplementary Figure 4 [file 41417_2022_567_MOESM4_ESM.pdf]
