# Supplementary material for: Single-cell discovery of the scene and potential immunotherapeutic target in hypopharyngeal tumor environment
Source: Cancer Gene Ther. 2022 Dec 2;30(3):462–71. doi: 10.1038/s41417-022-00567-x (PMC10014576; doi:10.1038/s41417-022-00567-x)
Supplement: Supplementary file 5 — Supplementary figure and table legends [file 41417_2022_567_MOESM5_ESM.docx]

**Supplementary figure and table legends**

**Figure S1. Quality control and partial functional enrichment of hypopharyngeal carcinoma** A. Diagrams of data filtering threshold value B. Before and after comparison pear plots of feature, count and mitochondrial level via quality control C. UMAP plots shows little batch effect between samples. D. No obvious batch effect between the samples; E. GO and KEGG enrichment of Eps-PEG10

**Figure S2. Significant role of BMP signaling pathway in the crosstalk network of CAFs and epithelial cells** A. River plots show BMP signaling is of specific between CAFs and most epithelial cells; B. Heatmap of BMP signaling pathway; C. Circle plot of BMP signaling pathway network; D. Heatmap of strength scores identifies signals that contribute most to efferent or afferent signaling in certain subgroups

**Figure S3. Potential hypopharyngeal cancer therapeutic target BMPR2** A. The positively correlation between BMPR2 and BMP4 under expression level in TCGA dataset B. Forest plot of Cox analysis of clinical data from BMPR2 and TCGA clinical data C. Expression of BMPR2 in each cell subtypes D. Selection basis of siRNA_BMPR2 E. The Venn plot of Targetscan predicting conserved miRNAs and GSE117558 significant different expressed miRNAs

**Figure S4. IHC Profiter results of 7 patients** A-G. Tissue staining of BMPR2. Bar plots represent the t-test results of negative rate of each tissue calculated by IHC Profiter. Method: t.test

**Supplementary Table.1 Metadata for this single-cell analysis**
